# Green Synthesis and Characterization of Silver Nanoparticles Using* Citrullus lanatus* Fruit Rind Extract

**DOI:** 10.1155/2017/8108504

**Published:** 2017-02-20

**Authors:** Michael Ndikau, Naumih M. Noah, Dickson M. Andala, Eric Masika

**Affiliations:** ^1^Chemistry Department, Kenyatta University, P.O. Box 43844, Nairobi 00100, Kenya; ^2^School of Pharmacy and Health Sciences, United States International University Africa, P.O. Box 14634, Nairobi 00800, Kenya; ^3^Multimedia University of Kenya, P.O. Box 30305, Nairobi 00100, Kenya

## Abstract

The wide-scale application of silver nanoparticles (AgNPs) in areas such as chemical sensing, nanomedicine, and electronics has led to their increased demand. Current methods of AgNPs synthesis involve the use of hazardous reagents and toxic solvents. There is a need for the development of new methods of synthesizing AgNPs that use environmentally safe reagents and solvents. This work reports a green method where silver nanoparticles (AgNPs) were synthesized using silver nitrate and the aqueous extract of* Citrullus lanatus* fruit rind as the reductant and the capping agent. The optimized conditions for the AgNPs synthesis were a temperature of 80°C, pH 10, 0.001 M AgNO_3_, 250 g/L watermelon rind extract (WMRE), and a reactant ratio of 4 : 5 (AgNO_3_ to WMRE). The AgNPs were characterized by Ultraviolet-Visible (UV-Vis) spectroscopy exhibiting a *λ*_max_ at 404 nm which was consistent with the spectra of spherical AgNPs within the wavelength range of 380–450 nm, and Cyclic Voltammetry (CV) results showed a distinct oxidation peak at +291 mV while the standard reference AgNPs (20 nm diameter) oxidation peak occurred at +290 mV, and Transmission Electron Microscopy (TEM) revealed spherical shaped AgNPs. The AgNPs were found to have an average diameter of 17.96 ± 0.16 nm.

## 1. Introduction

Nanotechnology is a new and emerging field of science that is bound to have tremendous impact on mankind by helping solve major challenges facing humanity in health and energy. This is due to the practical applications of metal nanoparticles in various areas such as medicine [1], chemical sensing, catalysis, and electronics [2]. Nanotechnology is the design, characterization, production and application of materials, devices and systems by controlling the shape and size of the nanometre scale [3]. Nanoparticles are particles that have a size of 1 to 100 nm in at least one dimension and possess unique physical and chemical properties due to their large surface area to volume ratio and smaller size [4].

There are two basic approaches used in nanoparticle synthesis: the top-down (communication and dispersion) approach and the bottom-up (nucleation and growth) approach. The decision on which method to adopt depends on the approach that can deliver the specified properties and on cost [3]. The three main methods of nanoparticle synthesis are physical, chemical and biological.

The physical method involves ball milling of bulk material to nanoscale size. Similarly, by physical vapour deposition in which a bulk material is vaporized by heat source followed by rapid condensation to form nanosized clusters that settle in the form of powder [5].

The chemical methods employed include sonochemical process (application of ultrasound to chemical reactions and processes); photochemical reductions (incident photons) are absorbed by reactant molecules to give excited molecules or free radicals, which undergo further reaction and sol-gel process.

The sol-gel process generally involves the use of metal alkoxides, which undergo hydrolysis and condensation polymerization reactions to give gels.

Plants and microbes such as bacteria, yeast, and fungi are used in the biological method of nanoparticle synthesis [6]. The physical and chemical methods of nanoparticle synthesis are costly; involve the use of high amounts of energy, toxic solvents and hazardous reagents [7]. The elaborate laboratory preparation of microbial cultures, the complex extraction and purification process of the synthesized nanoparticles make the microbial technique of nanoparticle synthesis expensive.

There is a need to develop new methods of synthesizing nanoparticles that are less costly, energy efficient and use nontoxic, environment-friendly renewable resources such as phytochemicals extracted from plants. This would definitely mean applying the “green chemistry” principles.

Green chemistry is the utilization of a set of principles that will help reduce the use and generation of hazardous substances during the manufacture and application of chemical products. Green chemistry aims to protect the environment not by cleaning up, but by inventing new chemical processes that do not pollute. It is a rapidly developing and important area in the chemical sciences [8].

Silver nanoparticles have greatly drawn the interest of many researchers because of their unique physical and chemical properties that make them find use in various applications. It has been reported that AgNPs are active against HIV-1 [9] and inhibit HIV-1 replication [10]. Studies have shown that AgNPs have potent antimicrobial activity [11].

Various plant extracts have been successfully used in the synthesis of silver nanoparticles. Some of the plant extracts used include* Camellia sinensis* (Green tea) leaf extract [12],* Cinnamomum camphora* leaf extract [13],* Aloe vera* extract [14],* Allium sativum* (Garlic) extract [15], and* Capsicum annum* (pepper) leaf extract [16].

Extensive review of available literature reveals that very few studies have been done to investigate the potential of agricultural wastes in synthesizing silver nanoparticles. The cheapest and environment-friendly source of silver nanoparticles would ideally use biomass that has no other useful competing applications. Agricultural wastes would offer such a source, for example, watermelon rind. The watermelon rind may potentially be used to synthesize AgNPs.

The* Citrullus lanatus* (watermelon) plant is an herbaceous creeping plant that originated from the Kalahari Desert of Southern Africa that produces 3 to 5 fruits weighing 3 to 8 kg each. The fruit can be round, oval or oblong having a light-green to dark green rind with or without stripes. The fruit has a fleshy centre that is usually red, yellow or white depending on the variety of the melon. The fleshy pulp of the fruit is rich in vitamins A and B_6_ and antioxidants lycopene and beta-carotene [17]. The seeds contain significant amounts of trace elements such as zinc, magnesium, and calcium [18]. The rind though edible, is usually discarded as an agricultural waste due to its unpleasant flavour. Studies have shown that the* Citrullus lanatus* (watermelon) rind extract contains polyphenols, tannins, alkaloids, flavonoids, and saponins [19]. It has been reported that polyphenols and flavonoids may be responsible for the synthesis of silver nanoparticles [20]. In this work, we show the potential of using* Citrullus lanatus* (watermelon) rind extract as a green method to synthesize AgNPs. The watermelon variety used in this study was the Charleston grey variety which is the most common sweet watermelon variety sold within Nairobi County, Kenya.

## 2. Materials and Methods

### 2.1. Preparation of Watermelon Rind Extract

**Figure 1:**
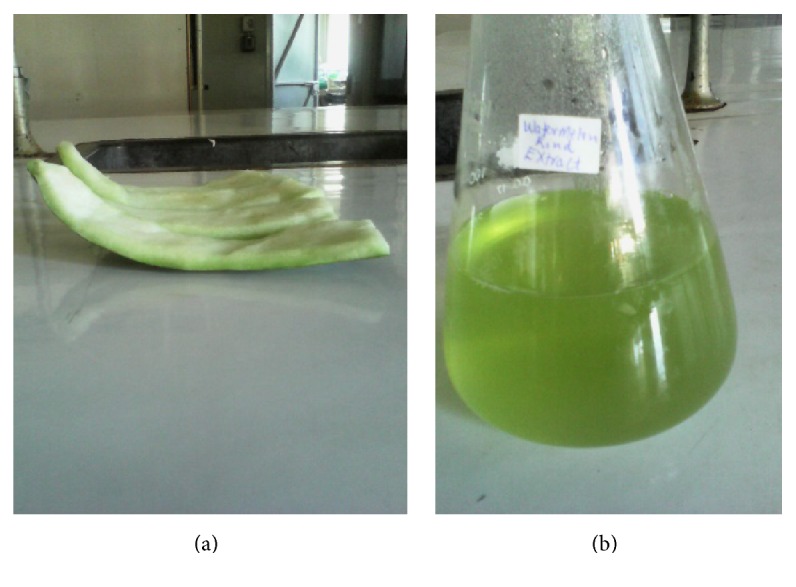
(a) Watermelon rind. (b) Watermelon rind extract.

One ripe watermelon fruit was thoroughly washed, rinsed with distilled water, and then using a clean sterilized knife cut into four quarter portions. The red coloured pulp in the interior of each watermelon portion was removed to obtain the watermelon rind as shown in Figure 1(a). The watermelon rinds were cut into small pieces (5 mm by 10 mm) using a sterile knife and placed inside a blender (Sunny blender) for crushing. Exactly, 100 grams of the crushed watermelon rind material was carefully weighed using an analytical balance and transferred into a clean 1000 mL conical flask and diluted with 400 mL of distilled water.

The 1000 mL conical flask was then placed in a shaking water-bath and heated at a temperature of 80°C for ten minutes in order to increase the yield of water soluble polyphenols in the watermelon rind extract. After the ten minutes, the 1000 mL conical flask was removed from the shaking water-bath and allowed to cool. The cold watermelon rind material in the 1000 mL conical flask was then filtered using Whatman number 1 filter paper. A light-green coloured filtrate was obtained as the watermelon rind extract as shown in Figure 1(b).

### 2.2. Preparation of Silver Nitrate Solution

The analytical grade silver nitrate (99.5% purity) was purchased from Sigma-Aldrich (USA) for use. About 0.1699 grams of analytical grade AgNO_3_ (99.5% Purity) was weighed using an analytical weighing balance and then transferred into a 1000 mL volumetric flask that contained 400 mL of distilled water. This was followed by stirring to ensure that all the solid AgNO_3_ dissolved. This was filled to the mark with distilled water. The concentration of silver nitrate was determined to be 0.001 M.

### 2.3. Synthesis of Silver Nanoparticles (AgNPs) Using the Watermelon Rind Extract (WMRE)

Silver nanoparticles were prepared by reacting silver nitrate solution (0.001 M) with the watermelon rind extract (250.0 g/L) as the reductant and stabilizing agent. The control method employed an already known and established method of synthesizing silver nanoparticles using trisodium citrate with a soluble silver salt [21]. This involved the reaction of a silver nitrate solution (0.001 M) with 1% trisodium citrate solution as the reductant and stabilizing agent. The second method was used for comparison purposes against the green method of using watermelon rind extract in silver nanoparticle synthesis.

### 2.4. Method Optimization

To obtain silver nanoparticles that are monodispersed with tunable size and morphology, various reaction parameters have to be controlled and optimized to achieve the desired results. These reaction parameters include the reaction temperature, pH of reaction mixture, concentration of reactants, and the ratio of the reactants [22].

#### 2.4.1. Optimization of the Ration of Reactants

To determine the optimal ratio of reactants (by volume), different ratios of reactants were used. Six samples were prepared in order to determine the optimal ratio (by volume) of the reactants. These samples were labeled as SG_1_, SG_4_, SG_5_, SG_6_, SG_7_, and SF_31_. The ratio (by volume) of the reactants: 1 mM AgNO_3_ to WMRE (watermelon rind extract) used to prepare the samples, were 1 : 1 for SG_1_, 1 : 4 for SG_4_, 1 : 5 for SG_5_, 2 : 3 for SG_6_, 3 : 5 for SG_7_, and 4 : 5 for SF_31_.

Sample SG_1_ was prepared using a volume ratio of 1 : 1 by adding 50 mL of 0.001 M AgNO_3_ solution to 50 mL of 250 g/L watermelon rind extract in a 250 mL conical flask. The pH of the reaction mixture was 5.92 and was adjusted to pH 10 using 0.1 M NaOH solution. This was done to promote the formation of AgNPs as an alkaline pH is known to favour formation of AgNPs [23]. The 250 mL conical flask containing the reaction mixture was then placed in a shaking water-bath at 80°C as higher temperatures used in preliminary experiments had shown an effect of increasing the maximum absorbance peak and the reaction rate. The colour of the reaction mixture changed from light-green to yellowish-brown after 35 minutes. Aqueous colloidal silver dispersion exhibits a characteristic yellow colour due to a phenomenon known as Surface Plasmon Resonance [5]. The 250 mL conical flask containing the sample was then removed from the shaking water-bath and allowed to cool to room temperature (25°C).

The WMRE-AgNPs sample was purified by centrifugation using Omega 6 Centrifuge at 5000 rpm for 30 minutes. The centrifugation of the sample was performed three times. The WMRE-AgNPs supernatant obtained was placed in clean glass vials. Exactly, 4 mL of the supernatant of the WMRE-AgNPs sample was placed in a quartz cuvette with a 1 cm path length and inserted in a UV-Vis spectrophotometer (CECIL CE 2041 2000 SERIES) in the wavelength range of 300–700 nm to obtain the UV-Visible spectra of the sample. All the other samples (SG_4_, SG_5_, SG_6_, SG_7_, and SF_31_) were prepared using the procedure outlined above, with the only difference being the use of different ratios of the reactants. These ratios of reactants represented different concentrations of the reactants. The ratio that gave the optimal reactant concentration was 4 : 5. The ideal concentration of the reactants was 4.44 × 10^−4^ M AgNO_3_ and 138.89 g/L of WMRE.

#### 2.4.2. Effect of Variation of Reaction Parameters on the WMRE-AgNPs Synthesis

*(1) Effect of Variation of Temperature*. 20 mL of 0.001 M AgNO3 were added to 25 mL of 250 g/L WMRE in a 250 mL conical flask and the pH of the reaction mixture was adjusted from 5.92 to 10 using 0.1 M NaOH solution. The 250 mL conical flask was then placed in a shaking water-bath at temperature of 40°C. The temperature of the reaction mixture was maintained at 40°C for 23 minutes in a shaking water-bath. The reaction mixture was removed from the shaking water-bath and allowed to cool to room temperature (25°C). The sample was purified by centrifugation using Omega 6 Centrifuge at 5000 rpm for 30 minutes. The centrifugation of the synthesized WMRE-AgNPs sample was performed three times. The supernatant of the WMRE-AgNPs obtained was placed in clean glass vials. The above procedure was repeated for temperature values of 50°C, 60°C, 70°C, and 80°C.

*(2) Effect of Variation of pH*. 20 mL of 0.001 M AgNO_3_ was added to 25 mL of 250 g/L WMRE in a 250 mL conical flask and the pH of the reaction mixture was adjusted from 5.92 to 6 using 0.1 M NaOH solution. The 250 mL conical flask was then placed in a shaking water-bath at temperature of 80°C. The temperature of the reaction mixture was maintained at 80°C for 23 minutes in a shaking water-bath. The reaction mixture was removed from the shaking water-bath and allowed to cool to room temperature (25°C). The sample was purified by centrifugation using Omega 6 Centrifuge at 5000 rpm for 30 minutes. The centrifugation of the synthesized WMRE-AgNPs sample was performed three times. The supernatant of the WMRE-AgNPs obtained was placed in clean glass vials. The above procedure was repeated for pH values of 8, 9, and 10.

*(3) Effect of Variation of Concentration of AgNO*_*3*_. 20 mL of 0.0001 M AgNO_3_ was added to 25 mL of 250 g/L WMRE in a 250 mL conical flask and the pH of the reaction mixture was adjusted from 5.92 to 10 using 0.1 M NaOH solution. The 250 mL conical flask was then placed in a shaking water-bath at temperature of 80°C. The temperature of the reaction mixture was maintained at 80°C for 23 minutes in a shaking water-bath. The reaction mixture was removed from the shaking water-bath and allowed to cool to room temperature (25°C). The sample was purified by centrifugation using Omega 6 Centrifuge at 5000 rpm for 30 minutes. The centrifugation of the synthesized WMRE-AgNPs sample was performed three times. The supernatant of the WMRE-AgNPs obtained was placed in clean glass vials. The above procedure was repeated for AgNO_3_ concentration values of 0.0002 M, 0.0004 M, 0.0008 M, and 0.001 M.

*(4) Effect of Variation of WMRE Concentration*. 20 mL of 0.001 M AgNO_3_ was added to 25 mL of 100 g/L WMRE in a 250 mL conical flask and the pH of the reaction mixture was adjusted from 5.92 to 10 using 0.1 M NaOH solution. The 250 mL conical flask was then placed in a shaking water-bath at temperature of 80°C. The temperature of the reaction mixture was maintained at 80°C for 23 minutes in a shaking water-bath. The reaction mixture was removed from the shaking water-bath and allowed to cool to room temperature (25°C). The sample was purified by centrifugation using Omega 6 Centrifuge at 5000 rpm for 30 minutes. The centrifugation of the synthesized WMRE-AgNPs sample was performed three times. The supernatant of the WMRE-AgNPs obtained was placed in clean glass vials. The above procedure was repeated for WMRE concentrations of 150 g/L, 200 g/L, and 250 g/L.

### 2.5. AgNPs Characterization

#### 2.5.1. UV-Visible Spectroscopy Characterization

The formation of AgNPs was confirmed using UV-Visible spectrophotometer (CECIL CE 2041 2000 SERIES). Exactly, 4 mL of the diluted supernatant of the WMRE-AgNPs sample was placed in a quartz cuvette with a 1 cm path length and inserted in a UV-Vis spectrophotometer in the wavelength range of 300–700 nm to obtain the UV-Visible spectra of the sample.

#### 2.5.2. Cyclic Voltammetry Characterization

The electrochemical activity of the synthesized nanoparticles was studied using a BASi EC Epsilon potentiostat complete with cell stand and data processor. The three-electrode system is composed of Glassy Carbon Electrode (GCE) as the working electrode, an Ag/AgCl (3 mol/L KCl) as the reference electrode, and a platinum wire as an auxiliary electrode.

AgNPs sample prepared using watermelon rind extract were immobilized on the Glassy Carbon Electrode surface by directly depositing 4 *μ*L of the AgNPs sample on the electrode surface and the solvent (water) allowed to evaporate at room temperature (25°C). Exactly, 10 mL of 0.05 M Phosphate buffer (pH 7.4) solution was placed in a voltammetric glass cell as the supporting electrolyte. The reference electrode was Ag/AgCl, the working electrode was the Glassy Carbon Electrode, and the counterelectrode was a platinum wire. The experimental parameters used were initial potential −1000 mV, switching potential +1100 mV, and final potential −1000 mV. The scan rates used ranged from 20 mV/s to 100 mV/s. The cyclic voltammogram obtained exhibited a distinct anodic peak at 290 mV at a scan rate of 100 mV/s. The electrochemical experiments were carried out at room temperature (25°C).

#### 2.5.3. Transmission Electron Microscopy Characterization

The electron microscopy of the AgNPs was performed using Zeiss Libra 120 TEM (120 kV). Samples of the WMRE-AgNPs, citrate-AgNPs, and standard reference AgNPs (20 nm diameter) were drop-coated on carbon-coated copper grids which had been placed on blotting paper and then allowed to dry in air for five minutes.

## 3. Results and Discussion

### 3.1. UV-Visible Spectroscopy Characterization AgNPs

UV-Visible spectroscopy of the AgNPs was performed to investigate the effect of variation of reaction parameters such as temperature, pH, and concentration of reactants on the synthesis of WMRE-AgNPs.

#### 3.1.1. Effect of Temperature on WMRE-AgNPs Synthesis

**Figure 2:**
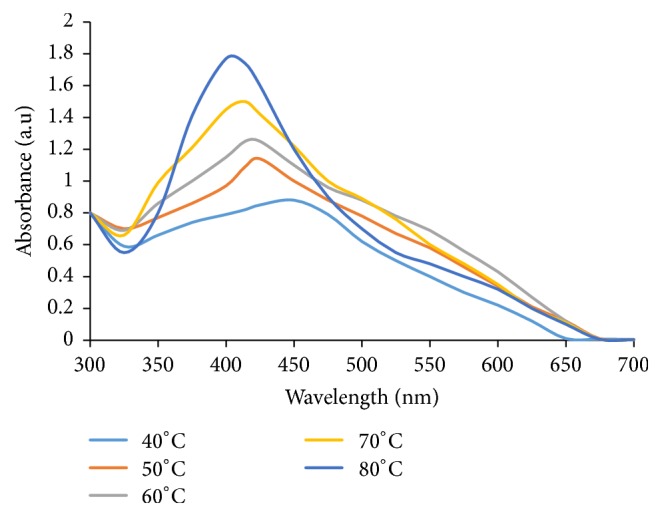
UV-Vis absorption spectra of WMRE-AgNPs sample SF_31_ showing the effect of variation of temperature on WMRE-AgNPs synthesis.

To investigate the effect of temperature on WMRE-AgNPs, different values of temperature were used in the synthesis of the AgNPs. The temperature range applied was 40°C to 80°C as described in Section 2.4.1. Exactly, 4 mL of the diluted supernatant of the WMRE-AgNPs sample was placed in a quartz cuvette with a 1 cm path length and inserted in a UV-Vis spectrophotometer (CECIL CE 2041 2000 SERIES) in the wavelength range of 300–700 nm to obtain the UV-Visible spectra of the sample as shown in Figure 2.

**Figure 3:**
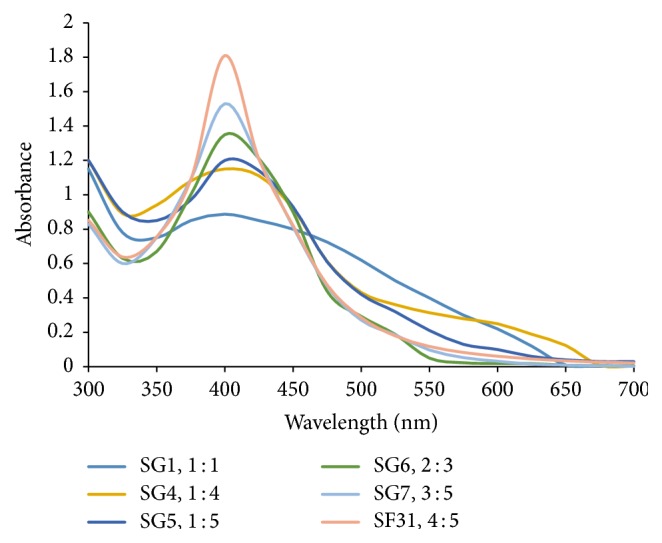
Showing spectra of WMRE-AgNPs samples SG1, SG4, SG5, SG6, SG7, and SF31 under optimized conditions of temperature (80°C) and pH 10.

The sample that exhibited a sharp, narrow intense peak of maximum absorption was SF_31_ as shown in Figure 3. This was indicative of the formation of relatively smaller sized AgNPs because the sharpness in absorbance peak depends on the size of the synthesized nanoparticle, [24]. Sample SF_31_ was the best sample obtained after optimizing the reaction conditions and was therefore chosen for all subsequent investigations of the reaction parameters.

A broad peak of less intensity was observed at 447 nm as shown in Figure 3 for the colloidal suspension obtained after heating the reaction mixture at 40°C. As the temperature was increased from 40°C to 80°C, a blue shift (hypsochromic shift) occurs due to reduction in the size of particles from large size to small size. There is also a disappearance of broadening. Broadening and a red shift are attributed to agglomeration or increase in size of the particles. The colloidal yellowish-brown solution, obtained after 23 minutes at 80°C, an absorption peak at 404 nm was observed which was consistent with UV-Vis spectra of spherical silver nanoparticles [5].

The optimum temperature required for the completion of reaction was found to be 80°C and the reaction time was 23 minutes.

It was observed that reduction rate of silver ions increased by the increasing of the temperature to 80°C. Similar results were reported by Amin et al. [25]. The sharpness in absorbance peak depends on the size of the synthesized nanoparticle [24].

#### 3.1.2. Effect of pH on WMRE-AgNPs Synthesis

**Figure 4:**
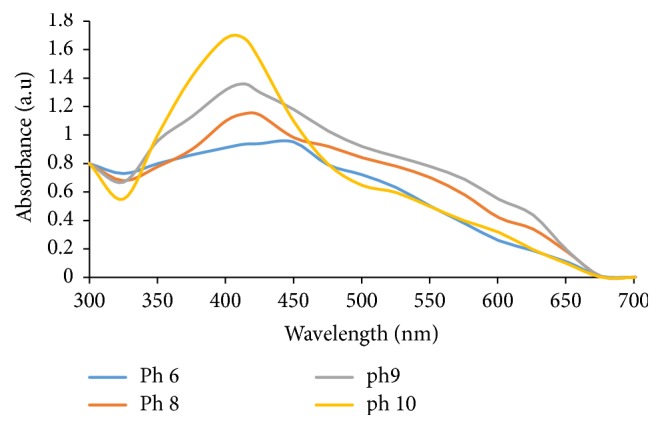
UV-Vis spectra of WMRE-AgNPs showing the effect of variation of pH on WMRE-AgNPs synthesis.

The synthesis of AgNPs by WMRE was performed over a pH range of 6–10.  Figure 4 shows the UV-Vis spectra of WMRE-AgNPs showing the effect of variation of pH on WMRE-AgNPs synthesis. At low pH (pH 6), the UV-Vis absorption peak is very broad as shown in Figure 4 (pH 6), this result is consistent with agglomerated AgNPs. The aggregation of AgNPs at low pH to form large nanoparticles is favoured over the nucleation [26].

There is a blue shift (hypsochromic shift) as the pH increases from pH 8 to pH 10 due to reduction in size of particles from large size to small size. A disappearance of the broadening of the peak is also observed. Broadening and red shift are attributed to agglomeration or increase in size of the particles. This indicates that the AgNPs size become smaller with increase in the pH. As the pH level increases, the bulk concentration of H^+^ ions decreases, resulting in a higher surface charge on the particle. Protonation and deprotonation surface reactions are used to obtain local surface charge which depends on particle size and pH [27]. At higher pH, the large number of phenolic functional groups available for silver binding facilitated a higher number of Ag^+^ ions to bind and subsequently form a large number of nanoparticles with smaller diameters. There was no formation of AgNPs at pH < 5, this phenomenon could be due to the instability of the nanoparticles at acidic pH [28]. This result confirmed the vital role played by pH in controlling the shape and size of the AgNPs. The optimum pH chosen for the synthesis was pH 10.

#### 3.1.3. Effect of AgNO_3_ Concentration on WMRE-AgNPs Synthesis

**Figure 5:**
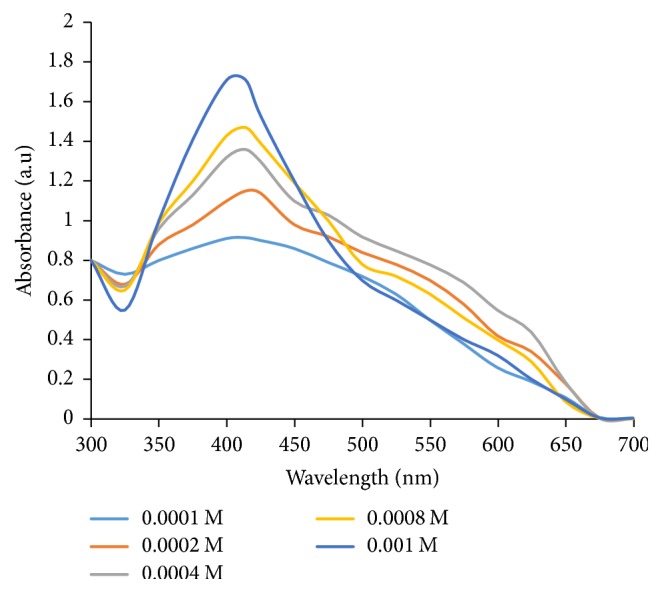
UV-Vis spectra of WMRE-AgNPs showing the effect of variation of AgNO_3_ concentration on WMRE-AgNPs synthesis.

The absorbance peak of the WMRE-AgNPs at low concentration of AgNO_3_ (0.0001 M) is broad and less intense as shown in Figure 5. This indicates that the AgNPs are agglomerated. However, as the AgNO_3_ concentration increases gradually from 0.0001 M to 0.001 M, the absorbance peak becomes sharper and intense and a blue shift occurs. This suggests that the WMRE-AgNPs get relatively smaller as the AgNO_3_ solution concentration increases to 0.001 M.

#### 3.1.4. Effect of WMRE Concentration on AgNPs Synthesis

**Figure 6:**
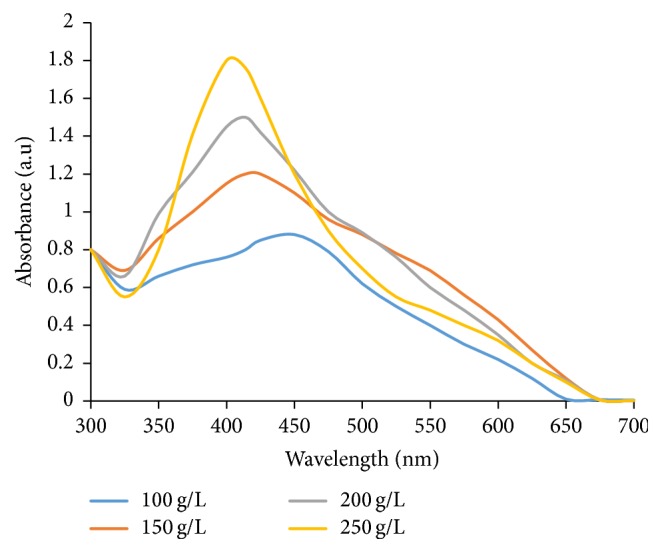
UV-Vis spectra of WMRE-AgNPs showing the effect of variation of WMRE concentration on WMRE-AgNPs synthesis.

The absorbance peak of the WMRE-AgNPs when the WMRE concentration was 100 g/L is broad and less intense as shown in Figure 6 and occurs at 456 nm. However, as the WMRE concentration increases gradually from 100 g/L to 250 g/L, the absorbance peak becomes more narrow and intense. There is a gradual blue shift as the WMRE concentration increases from 100 g/L to 250 g/L (Figure 6). A blue shift coupled with sharp and intense absorbance peak is associated with a reduction in the size of AgNPs. Since at lower extract concentration a smaller number of nucleation sites would be present so more reduction would take place at one nuclei leading to formation of a bigger particle.

However, it is also possible that at higher concentrations the polyphenols in the WMRE had effectively reduced the Ag^+^ ions to Ag^0^ and provided enough capping agent for the stabilization of the synthesized nanoparticles through steric hindrance thus preventing their aggregation [29]. These results are in agreement with those obtained by Subramanian et al. [30].

**Figure 7:**
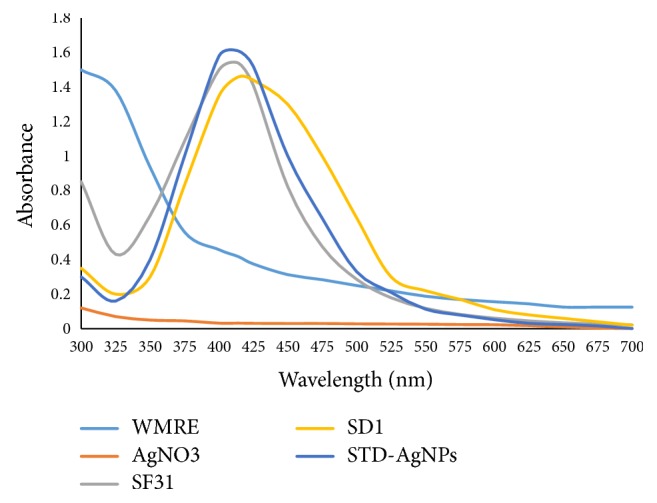
UV-Vis spectra of watermelon rind extract (WMRE), AgNO_3_ (0.001 M) solution, sample SF_31_ (WMRE-AgNPs), and sample SD_1_ (citrate-AgNPs).

The reactants and products of the AgNPs synthesis were separately placed in quartz cuvette and their UV-Visible spectra obtained. Briefly, 4 mL of the diluted supernatant of the WMRE-AgNPs sample was placed in a quartz cuvette with a 1 cm path length and inserted in a UV-Vis spectrophotometer (CECIL CE 2041 2000 SERIES) in the wavelength range of 300–700 nm to obtain the UV-Visible spectra of the sample as shown in Figure 7.

It can be observed from Figure 7 that sample SF_31_ had a narrow absorption peak with the wavelength of maximum absorption at 404 nm compared with that of sample SD_1_ (citrate-AgNPs) which was broad with a wavelength of maximum absorption at 437 nm. This suggested that the AgNPs in sample SF_31_ were of a relatively smaller size than those in sample SD_1_ because small size AgNPs absorb and scatter electromagnetic radiation at shorter wavelengths than larger size AgNPs [31]. Also, Figure 7 shows that the maximum absorption peaks of samples SF_31_ and SD_1_ are not due to unreacted watermelon rind extract and unreacted AgNO_3_ 0.001 M solution.

### 3.2. Cyclic Voltammetry Characterization

The electrochemical detection of metal nanoparticles can be carried out in two ways: immobilizing the nanoparticles on the electrode surfaces or direct detection of the nanoparticles hitting the surface of the electrode [32].

**Figure 8:**
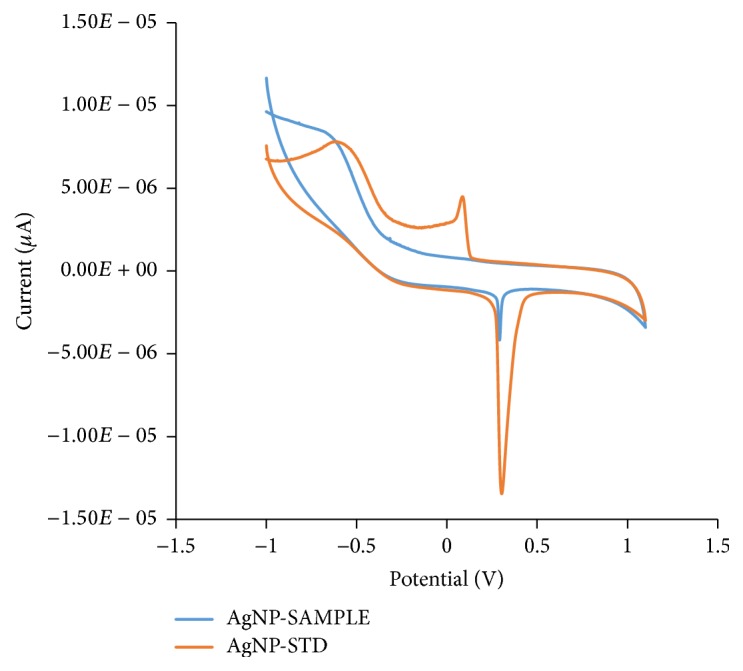
Overlay of cyclic voltammograms of standard reference AgNPs (0.02 mg/mL, 20 nm AgNPs colloidal dispersion) and WMRE-AgNPs.

Cyclic Voltammetry experiments on the standard AgNPs (0.02 mg/mL, 20 nm AgNPs colloidal dispersion) and AgNPs samples prepared using watermelon rind extract as the reductant and stabilizing agent were carried out to confirm whether AgNPs were present in the samples prepared using the green chemistry method. The cyclic voltammogram of standard AgNPs exhibited distinct oxidation and reduction peaks at +290 mV and +100 mV as shown in Figure 8.

The cyclic voltammogram of AgNPs sample prepared using watermelon rind extract as the reductant shows a distinct oxidation peak at +291 mV (as shown in Figure 8). Different sized AgNPs exhibit different voltammetric profiles [32]. The slight difference in the position of oxidation peak potential of the standard AgNPs (290 mV) with a diameter of 20 nm and WMRE-AgNPs (291 mV) with an average diameter of 17.96 ± 0.16 could be due to a difference in the sizes of AgNPs.

### 3.3. Transmission Electron Microscopy (TEM)

**Figure 9:**
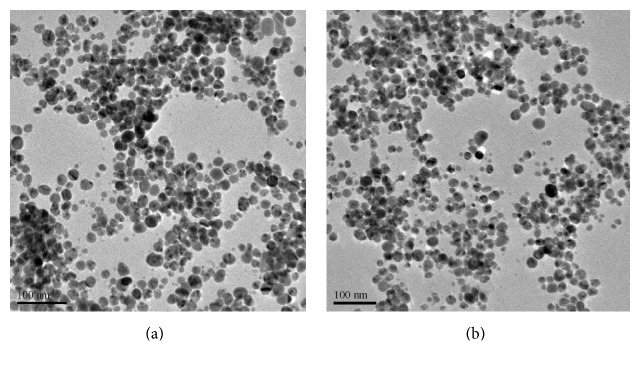
(a) TEM micrograph of WMRE-AgNPs. (b) TEM micrograph of citrate-AgNPs.

The WMRE-AgNPs formed were spherical in shape with an average diameter of 17.96 ± 0.16 nm while the citrate-AgNPs were spherical with an average diameter of 36.96 ± 0.51 nm as shown in Figures 9(a) and 9(b). The data from the TEM was analyzed using one-way ANOVA to obtain the average diameter of the AgNPs. Thus, the watermelon rind aqueous extract as a reductant yielded smaller AgNPs than those obtained using trisodium citrate as the reductant and stabilizing agent. Similar results have been reported in literature where different green methods have been used to synthesize spherical shaped AgNPs with an average diameter ranging between 20 and 34 nm [33–36]. This indicates that* Citrullus lanatus* fruit rind served as a good reductant and a capping agent in the synthesis of AgNPs.

## 4. Conclusions

We have successfully developed a one-step green synthesis protocol that utilizes* Citrullus lanatus* fruit rind as a reductant and a capping or stabilizing agent in the synthesis of AgNPs through green chemistry. The optimum conditions for this process were a temperature of 80°C, pH 10, and reactant ratio 4 : 5 (AgNO_3_ 0.001 M and 250 g/L, resp.). This method yields stable, spherical silver nanoparticles with an average hydrodynamic diameter of 17.96 ± 0.16 nm.
